# Metabolite differences in the medial prefrontal cortex in schizophrenia patients with and without persistent auditory verbal hallucinations: a ^1^H MRS study

**DOI:** 10.1038/s41398-022-01866-5

**Published:** 2022-03-23

**Authors:** Qianjin Wang, Honghong Ren, Chunwang Li, Zongchang Li, Jinguang Li, Hong Li, Lulin Dai, Min Dong, Jun Zhou, Jingqi He, Joseph O’Neill, Yanhui Liao, Ying He, Tieqiao Liu, Xiaogang Chen, Jinsong Tang

**Affiliations:** 1 Department of Psychiatry, and National Clinical Research Center for Mental Disorders, The Second Xiangya Hospital of Central South University, Changsha, Hunan China; 2 Hunan Key Laboratory of Psychiatry and Mental Health, Changsha, Hunan China; 3 Department of Radiology, Hunan Children’s Hospital, Changsha, China; 4 Department of Psychiatry, the First Affiliated Hospital of Zhengzhou University, Zhengzhou, China; 5 Guangdong Mental Health Center, Guangdong Provincial People’s Hospital, Guangdong Academy of Medical Sciences, Guangzhou, Guangdong China; 6 Division of Child and Adolescent Psychiatry, UCLA Semel Institute for Neuroscience and Human Behavior, Los Angeles, CA USA; 7 Department of Psychiatry, Sir Run-Run Shaw Hospital, School of Medicine, Zhejiang University, Hangzhou, China

**Keywords:** Biomarkers, Diseases

## Abstract

Studies of schizophrenia (SCZ) have associated auditory verbal hallucinations (AVH) with structural and functional abnormalities in frontal cortex, especially medial prefrontal cortex (mPFC). Although abnormal prefrontal network connectivity associated with language production has been studied extensively, the relationship between mPFC dysfunction (highly relevant to the pathophysiology of SCZ) and AVH has been rarely investigated. In this study, proton magnetic resonance spectroscopy was used to measure metabolite levels in the mPFC in 61 SCZ patients with persistent AVH (pAVH), 53 SCZ patients without AVH (non-AVH), and 59 healthy controls (HC). The pAVH group showed significantly lower levels of *N*-acetyl-aspartate + *N*-acetyl-aspartyl-glutamate (tNAA) and glutamate + glutamine (Glx), compared with the non-AVH (tNAA: *p* = 0.022, Glx: *p* = 0.012) and HC (tNAA: *p* = 0.001, Glx: *p* = 0.001) groups. No difference was found in the levels of tNAA and Glx between non-AVH and HC. The levels of tNAA and Glx in the mPFC was negatively correlated with the severity of pAVH (tNAA: *r* = −0.24, *p* = 0.014; Glx: *r* = −0.30, *p* = 0.002). In conclusion, pAVH in SCZ patients might be related to decreased levels of tNAA and Glx in the mPFC, indicating that tNAA or Glx might play a key role in the pathogenesis of pAVH.

## Introduction

Auditory verbal hallucinations (AVH), defined as vocal perceptual experiences that occurs without any corresponding external stimuli [1], are a core symptom of schizophrenia (SCZ) that affect 60–80% of SCZ patients [2–4]. Persistent AVH (pAVH) are a type of AVH that can last for more than one year despite the use of two different antipsychotics [5]. AVH bring a huge burden to patients and are usually related to social and occupational dysfunction, as well as poor prognosis [6–8]. Although antipsychotics can quickly reduce the frequency and severity of AVH for most patients [9], there are still 25–30% of SCZ cases that are chronically resistant to traditional antipsychotics [4, 10].

The medial prefrontal cortex (mPFC) forms part of the mesocorticolimbic dopamine (DA) system [11], which is believed to be engaged in the integration of information from many cortical and subcortical regions and in the aggregation of updated information. It is also involved in cognitive processes of reality monitoring, emotional regulation, motivation, and social skills [12–14]. Reality monitoring is a cognitive ability to distinguish between real and imagined information [13], but it is usually abnormal in patients with SCZ who hallucinate (including those with AVH) [13, 14]. Neuroimaging technologies have also provided evidence that AVH are usually associated with abnormal structure and neuro-metabolism of frontal and temporal areas [10, 15–17], which are engaged in multiple functions, especially language production and perception [18–20]. Previous studies have shown that patients with pAVH have smaller gray matter volume [21] and lower functional connectivity [22] in the mPFC compared to patients without AVH and HC. However, to date, there is no report on the relationship between the levels of metabolites in the mPFC and pAVH in SCZ patients.

^1^H-MRS (proton magnetic resonance spectroscopy) is a standard non-invasive technique used to measure the levels of metabolites in the human brain [2, 23–25]. Previous ^1^H-MRS studies on patients with SCZ showed abnormalities in the levels of *N*-acetyl-compounds [17, 23, 26–28]. The *N*-acetyl-compounds are *N*-acetyl-aspartate (NAA) and *N*-acetyl-aspartyl-glutamate (NAAG). Due to highly overlapping spectral peaks, NAA and NAAG are poorly separated by ^1^H MRS at 1.5 or 3 T, hence they are usually measured together as the sum tNAA = NAA + NAAG. In SCZ, MRS has also detected abnormalities in levels of glutamate (Glu) or glutamine (Gln) [2, 24, 26]. Glu and Gln also have overlapping signals and are often measured as the sum Glx = Glu + Gln. Abnormalities have also been observed in glycerophosphocholine + phosphocholine (GPC + Pecha) [26, 27], creatine + phosphocreatine (PCr) [27, 28], and myo-inositol (mI) [26, 29], or of the ratios of these metabolites to Cr + PCr or GPC + PCh in SCZ with MRS. Most of these studies focused on the dorsolateral prefrontal cortex (DLPFC) [2, 17], anterior cingulate cortex (ACC) [24, 30], thalamus [31], and hippocampus [4, 25], but not on the mPFC.

In this study, we aim to investigate the connection between pAVH and MRS metabolites in the mPFC. For this purpose, in pAVH, non-AVH, and HC participant groups, we compared metabolite levels, as well as demographic and clinical variables. We also analyzed correlations between the severity of pAVH and the levels of metabolites in the mPFC. The main focus was on tNAA and Glx. Based on previous MRS studies of frontal cortex in patients with SCZ [32, 33], we hypothesized that tNAA in mPFC would be decreased in SCZ patients with pAVH compared to non-AVH patients and to HC. We further hypothesized that the tNAA level would be negatively correlated with the severity of pAVH. Given the evidence of decreased levels of Glx in SCZ patients [2], we also hypothesized that the Glx level would be decreased in SCZ patients with pAVH. We also explored group-differences in other metabolites in the mPFC and their correlations with the severity of pAVH.

## Materials and methods

This study was approved by the Ethics Committee of the Second Xiangya Hospital, Central South University (No. S006, 2018), and was conducted in accordance with the Declaration of Helsinki. After being fully informed of the benefits and potential risks of the study, the participants provided written informed consent.

### Participants

121 SCZ patients were recruited from the Psychiatric Clinic at the Second Xiangya Hospital of Central South University in China. Contemporaneously, 60 healthy controls (HC) were recruited via local community advertisements. All the patients were diagnosed with SCZ per DSM-IV-TR by two trained senior psychiatrists using Mini-International Neuropsychiatric Interviews [34]. The inclusion criteria were: (1) Han Chinese aged between 16 and 45 years; (2) right-handed; (3) normal hearing and intelligence; (4) no history of substance abuse; (5) no history of major medical or neurological diseases or trauma. The Positive and Negative Symptom Scale (PANSS) was used to evaluate the severity of the patients’ current symptoms [35], and the P3 item in PANSS was used to evaluate the severity of AVH [36]. The patients were divided into two subgroups according to the presence of treatment-resistant AVH. Sixty-one patients had a score of the P3 hallucination item in PANSS of >3 (i.e., presence of pAVH) and were assigned to the pAVH group, and 60 patients had a P3 hallucination score of =1 (i.e., absence of AVH) and were assigned to the non-AVH group [17, 37]. Treatment-resistant AVH are defined as AVH that are persistent despite the use of at least two antipsychotic drugs at sufficient dosage for over 6 weeks [21, 38]. All the patients enrolled in the study were treated for at least one year at the same antipsychotic dose but still had disabling symptoms due to resistance. No HC met the diagnostic criteria for any DSM-IV-TR mental disorder, nor had a history of early mental disorder or family history of mental illnesses.

### MRS data acquisition

**Fig. 1:**
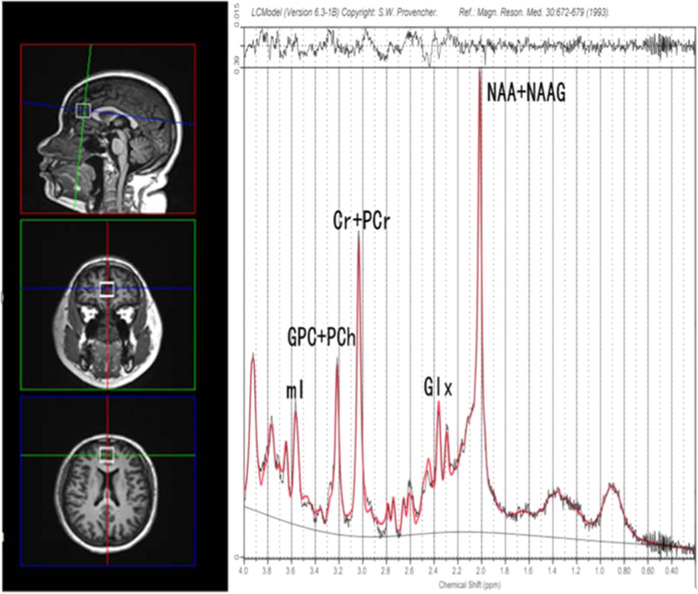
The location and sample spectrum of the voxel of interest in the medial prefrontal cortex (mPFC). The voxel dimension is 20 × 20 × 20 mm^3^.

MRS was acquired from all the participants within 24 h after enrollment to assess metabolites levels in the mPFC. All MRI data were acquired using a 3.0 T MRI scanner (Siemens Skyra, Munich) with a 16-channel headcoil at the Magnetic Imaging Center of Hunan Children’s Hospital. During scanning, foam pads and earplugs were used to restrain head movement and to attenuate noise. Anatomical T1-weighted MRI data were acquired using a 3D magnetization-prepared rapid acquisition gradient-echo (3D MPRAGE) sequence with the following parameters: TR/TE = 2530 ms/2.33 ms, flip angle = 7°, field of view = 256 × 256 mm, slice thickness = 1 mm, number of excitations (NEX) = 1, gap = 0 mm, and number of slices = 192. The data acquired were used for tissue-segmentation of MRS voxels. In order to obtain a consistent location of voxels of interest (VOI), all participants were positioned by the same investigator according to easily identifiable anatomical landmarks: the mPFC VOI (20 × 20 × 20 mm^3^) was placed anterior to the genu corpus callosum and parallel to the anterior-to-posterior commissure (AC-PC) line. ^1^H-MRS spectra were acquired using the standard point-resolved spectroscopy sequence (PRESS; svs_se, TR/TE = 3000 ms/30 ms, spectral bandwidth = 1200 Hz, NEX = 80). A target VOI was positioned in the gray matter of the mPFC using coronal, sagittal and transverse images (Fig. 1). Pre-saturation pulses of variable power radiofrequency pulses with optimized relaxation delays (VAPOR) were used for suppression of the water signal. Water unsuppressed spectra were acquired in an identical voxel. Spectra without water suppression were acquired with parameters identical to those of water suppressed spectra, except that for disabled water suppression was disabled and NEX = 8. The spectral processing function of the Siemens Spectral application (Syngo B17, Germany) was used to test the preliminary quality of the spectra. Spectra with significant baseline drift were excluded, and the patients were scanned again with their consent. The SPM12 segmentation tool (FIL Wellcome Department of Imaging Neuroscience, London, UK) was used to segment the T1-weighted MRI images into gray matter (GM), white matter (WM), and cerebrospinal fluid (CSF). We also collected information about the name, dose, and duration of the antipsychotics used by the patients, and the doses of the antipsychotics were converted to equivalent milligrams of chlorpromazine (CPZ). All of the patients were on antipsychotic medications on the day of the MRI scan, and their medications were not adjusted prior to the scan.

### MRS data analyses

The ^1^H-MRS spectra were fit with the LCModel version 6.3-1B [39] at the Second Affiliated Hospital of Shantou University Medical College, Guangdong, China. Supplementary Table 2 shows a standard basis set of metabolites for analysis. Raw spectra were fit to major resonances of common metabolites including NAA, NAAG, Glu, Gln, Cr, PCr, GPC, PCh, mI, etc. (Fig. 1), with the unsuppressed water signal in mPFC used as an internal reference for absolute metabolite quantification. To ensure data reliability, only metabolite spectra that met the following criteria were retained: (1) the Cramer–Rao lower bound (CRLB) < 20%; (2) the full-width at half-maximum (FWHM) ≤ 0.1 ppm; and (3) the signal-to-noise ratio (SNR) ≥ 20.

Per the LCModel Manual, each metabolite level was normed to the water reference and corrected for voxel CSF-content using the following equation:

$$M_{{\mathrm {corr}}} = M\frac{{35,880{{{\mathrm{WM}}}} + 43,300{{{\mathrm{GM}}}} + 55,556{{{\mathrm{CSF}}}}}}{{35,880(1 - {{{\mathrm{CSF}}}})}} = M\frac{{{\mathrm {WM}} + 1.21{\mathrm {GM}} + 1.55{\mathrm {CSF}}}}{{(1 - {{{\mathrm{CSF}}}})}}$$

where *M*_corr_ is the corrected metabolite value, *M* is the uncorrected value, and WM, GM and CSF are the volume fractions of white matter, gray matter, and cerebrospinal fluid in the MRS voxel. The denominator factor 35,880 undoes the LCModel assumption built into the value of *M* that the MRS voxel consists of pure white matter with a water concentration of 35,880 mol/m^3^. The numerator additionally uses the pure gray matter (43,300 mol/m^3^) and pure cerebrospinal fluid (55,556 mol/m^3^) water concentrations for a better estimate of the true voxel water concentration accounting for the voxel tissue-composition. Finally, although a complete correction of metabolite levels for partial-voluming is not feasible, the factor 1/(1 − CSF) corrects for the CSF-content of the voxel. We did not correct metabolite levels for relaxation effects.

### Statistical analysis

All the statistical analyses were performed using the software SPSS 26 (SPSS Inc., Chicago, IL, USA). The normality of each variable was tested using the Kolmogorov–Smirnov test prior to the analyses. Demographic endpoints were compared across groups with the use of either one-way Chi-squared test, analysis-of-variance (ANOVA), or Student’s *t*-test, when appropriate. As demographic and clinical data were not normally distributed, we used a nonparametric test, Mann–Whitney *U* test, for inter- group comparison. Univariate covariance analysis (ANCOVA) was used to compare the level of metabolites among the three groups. When the difference was significant in the above comparisons, post hoc tests were then performed using Bonferroni correction for multiple comparisons. Partial correlation analysis was used to investigate the relationship between the severity of pAVH and the level of metabolites in the mPFC. The threshold of statistical significance was set at *p* = 0.05 (two-tailed).

## Results

### General Information

The final analysis included 61 SCZ patients with pAVH, 53 SCZ patients with non-AVH, and 59 HC. Data for 7 non-AVH patients (3 were lost to follow-up, 2 had contraindications to MR, and 2 had abnormal scans) and 1 HC (contraindication to MRI) were excluded. No significant difference was found in age, gender, smoking and drinking status, age at disease onset, duration of disease, or CPZ equivalent dosage among the three groups (Table 1). The age range of the pAVH group, non-AVH group and HC group was 17–38 years, 16–42 years, and 18–43 years, respectively. Gender differences regarding the level of metabolites in the mPFC are detailed in Supplementary Table 1. The HC had significantly higher level of education than the patients with pAVH (*p* < 0.001) and those with non-AVH (*p* = 0.012). Therefore, ANCOVA was performed for the level of metabolites, with the level of education as a covariate.

**Table 1:** Demographic information, smoking and drinking status and clinical characteristics of participants.

Characteristics	Healthy Control (HC) (*n* = 59)	Patients(*n* = 114)			Significance		
		pAVH (*n* = 61)	non-AVH (*n* = 53)	3 groups	HC vs. non-AVH *p* value	HC vs. pAVH	pAVH vs. non-AVH
Gender (M/F), *n*	25/34	31/30	23/30	*χ*² = 1.02 (0.60)	0.91	0.35	0.43
Age (y), (*M* ± SD)	27.25 ± 6.10	25.43 ± 5.41	27.19 ± 6.19	*F* = 1.84 (0.16)	1.00	0.27	0.34
Education (y), (*M* ± SD)	14.44 ± 2.66	11.99 ± 3.38	12.79 ± 2.82	*F* = 10.48(<0.001)^**^	0.012^*^	<0.001^**^	0.46
Smoke (yes/no), *n*	9/50	9/52	13/40	*χ*² = 2.27 (0.32)	0.22	0.94	0.19
Drinker (yes/no), *n*	2/57	0/61	1/52	*χ*² = 2.03 (0.36)	0.62	0.15	0.28
Age at disease onset (y), (*M* ± SD)	–	20.92 ± 4.92	21.38 ± 5.02	–	–	–	*U* = 1532 (0.63)
Illness duration (y), (*M* ± SD)	–	7.02 ± 4.65	5.99 ± 4.15	–	–	–	*U* = 1369 (0.27)
PANSS-T, (*M* ± SD)	–	60.12 ± 14.02	51.34 ± 17.01	–	–	–	*U* = 964 (<0.001)^**^
PANSS-P, (*M* ± SD)	–	16.28 ± 3.97	10.21 ± 3.43	–	–	–	*U* = 369.5 (<0.001)^**^
PANSS-N, (*M* ± SD)	–	15.80 ± 5.83	14.09 ± 7.48	–	–	–	*U* = 1202 (0.025)^*^
PANSS-G, (*M* ± SD)	–	27.70 ± 7.03	27.04 ± 8.15	–	–	–	*U* = 1430.5 (0.36)
P3 hallucination item of PANSS, (*M* ± SD)	–	5.10 ± 0.75	1.00 ± 0.00	–	–	–	*U* = 0.000 (<0.001)^**^
CPZ equivalent(mg/d), (*M* ± SD)	–	730.52 ± 342.87	595.93 ± 402.90	–	–	–	*T* = 1.87 (0.06)

*M* mean, *SD* standard deviation, *n* number, *M/F* male/female, *pAVH* persistent auditory verbal hallucinations, *non-AVH* without auditory verbal hallucinations, *HC* health control, *PANSS* Positive and Negative Symptoms Scale, *PANSS-T* PANSS total score, *PANSS-P* PANSS positive score, *PANSS-N* PANSS negative score, *PANSS-G* PANSS general psychopathology score, *CPZ* chlorpromazine.

*p < 0.05; **p < 0.01.

With regard to the clinical symptoms, the total PANSS score (PANSS-T, *p* < 0.001), score for positive symptoms (PANSS-P, *p* < 0.001), score for negative symptoms (PANSS-N, *p* = 0.025), and score for P3 hallucinations (*p* < 0.001) in patients with pAVH were significantly higher than the corresponding scores in patients with non-AVH. However, no significant difference in the PANSS general psychopathological symptom score (PANSS-G) was found between the two patient groups (Table 1).

### Quality of ^1^H-MRS spectra

There was no significant difference in SNR, FWHM, GM, WM, and CSF among the three groups (Table 2). The CRLB values of metabolites in mPFC in the three groups were all <20%.

**Table 2:** Quality of MRS data of patients and healthy controls.

Variable	*M* ± SD			ANOVA	HC vs. non-AVH	HC vs. pAVH	pAVH vs. non-AVH
	HC	pAVH	Non-AVH				
SNR	22.09 ± 5.73	20.77 ± 4.41	22.06 ± 4.06	*F* = 1.45 (0.24)	1.00	0.41	0.47
FWHM (ppm)	0.07 ± 0.02	0.08 ± 0.03	0.07 ± 0.03	*F* = 0.25 (0.78)	1.00	1.00	1.00
GM	0.56 ± 0.08	0.54 ± 0.07	0.54 ± 0.09	*F* = 0.79 (0.46)	0.89	0.79	1.00
WM	0.38 ± 0.08	0.39 ± 0.07	0.39 ± 0.09	*F* = 0.94 (0.39)	0.85	0.62	1.00
CSF	0.07 ± 0.01	0.06 ± 0.01	0.07 ± 0.01	*F* = 1.23 (0.30)	1.00	0.40	0.82

*M* mean, *SD* standard deviation, *ANOVA* one-way analysis of variance, *pAVH* persistent auditory verbal hallucinations, *non-AVH* without auditory verbal hallucinations, *HC* health control, *FWHM* full width half maximum, *ppm* parts-per-million, *SNR* signal to noise ratio, *GM* gray matter, *WM* white matte, CSF cerebrospinal fluid.

### Levels of metabolites in the mPFC

**Fig. 2:**
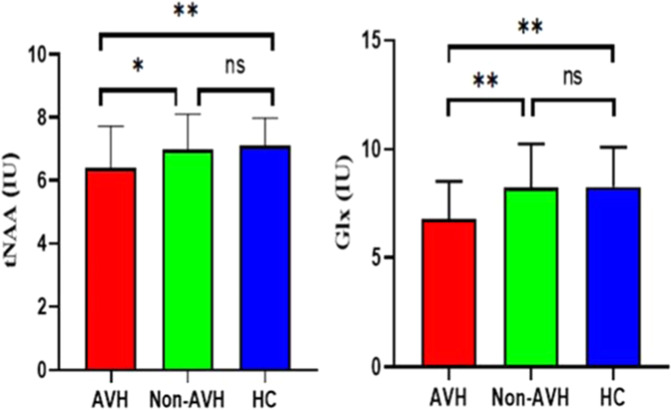
Differences in brain metabolites in the medial prefrontal cortex (mPFC) among the three groups of participants. The patients with persistent auditory verbal hallucinations (pAVH) displayed significant reductions in levels of Glx and tNAA, compared to patients without AVH (non-AVH) (tNAA: *p* = 0.022, Glx: *p* = 0.012) and healthy controls (HC) (tNAA: *p* = 0.001, Glx: *p* = 0.001). No significant inter-group differences were found between non-AVH and HC. Error bars represent standard deviations. IU institutional units. ns not significant. *p < 0.05, **p < 0.01.

The levels of metabolites were compared among the three groups using ANCOVA, with age, gender, education level as covariates. The ANCOVA was used to compare the levels of metabolites between the two patient groups with age, gender, education level, PANSS-P score, PANSS-N score, and CPZ equivalent dose as covariates. The ANCOVA analysis found significant differences in the levels of tNAA (*F* = 7.56, *p* = 0.001) and Glx (*F* = 9.90, *p* < 0.001) among the three groups. Further post-hoc comparisons showed that the levels of tNAA and Glx were significantly lower in patients with pAVH than in patients with non-AVH (tNAA: *p* = 0.022, Glx: *p* = 0.012) and HC (tNAA: *p* = 0.001, Glx: *p* = 0.001); however, the difference was not significant between patients with non-AVH and HC (tNAA: *p* = 1.00, Glx: *p* = 1.00). In addition, the levels of Cr + PCr (*F* = 1.54, *p* = 0.22), GPC + PCh (*F* = 0.06, *p* = 0.94) or mI (*F* = 0.65, *p* = 0.52) were not significantly different among the three groups. All the above comparisons were adjusted using Bonferroni correction (Table 3 and Fig. 2).

**Table 3:** Metabolite concentrations and CRLB in the mPFC in SCZ patients and healthy controls^a^.

Variable	*M* ± SD			ANCOVA^b^	HC vs. non-AVH	HC vs. pAVH	pAVH vs. non-AVH^c^
	HC	pAVH	non-AVH				
tNAA	7.10 ± 0.88	6.37 ± 1.33	6.98 ± 1.13	*F* = 7.56 (0.001)^**^	1.00	0.001^**^	0.022^*^
Glx	8.27 ± 1.82	6.79 ± 1.75	8.24 ± 2.00	*F* = 9.90 (<0.001)^**^	1.00	0.001^**^	0.012^*^
Cr + PCr	5.29 ± 0.65	5.13 ± 0.89	5.09 ± 0.66	*F* = 1.54 (0.22)	0.30	0.53	0.10
GPC + PCh	1.11 ± 0.18	1.07 ± 0.27	1.11 ± 0.17	*F* = 0.06 (0.94)	1.00	0.70	0.36
mI	4.45 ± 1.10	4.59 ± 2.16	4.72 ± 1.37	*F* = 0.65 (0.52)	0.79	1.00	0.52

The three columns on the right-hand side are results of post-hoc tests, Bonferroni corrected.

*M* Mean, *SD* standard deviation, *pAVH* persistent auditory verbal hallucinations, non-*AVH* without auditory verbal hallucinations, *HC* health control, *ANCOVA* analysis of covariance, *tNAA* N-acetyl-aspartate (NAA) + N-acetyl-aspartyl-glutamate (NAAG), *Glx* glutamate (Glu) + glutamine (Gln), *Cr* *+* *PCr* creatine + phosphocreatine; *GPC* *+* *PCh* glycerophosphocholine + phosphocholine, *mI* myo-inositol.

*p < 0.05; **p < 0.01.

^a^All metabolite levels are in institutional units (IU).

^b^The levels of metabolites were compared among the three groups using ANCOVA, with age, gender, and education level as covariates.

^c^The ANCOVA was used to compare the levels of metabolites between the two patient groups with age, gender, education level, PANSS-P score, PANSS-N score, and CPZ equivalent dose as covariates.

### Correlation of level of metabolites with the severity of pAVH

**Fig. 3:**
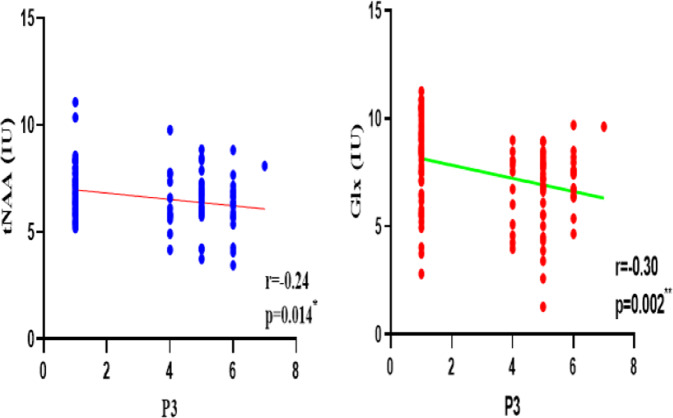
The relationship between the abnormality of brain metabolites in medial prefrontal cortex (mPFC) and the severity of persistent auditory verbal hallucination (pAVH) in patients with schizophrenia. The relationship between the severity of pAVH (measured by the P3 hallucination item in PANSS) and the level of tNAA (*r* = −0.24, *p* = 0.014) and Glx (*r* = −0.30, *p* = 0.002) in mPFC. IU institutional units. *p < 0.05, **p < 0.01.

Partial correlations were used to investigate associations between the level of metabolites in the mPFC and the severity of pAVH (the score of P3 hallucination item in PANSS score) after controlling for age, gender, education level, score of PANSS-N and CPZ equivalent dosage. After Bonferroni correction, the levels of tNAA and Glx in MCPc were significantly correlated with the severity of pAVH (tNAA: *r* = −0.24, *p* = 0.014; Glx: *r* = −0.30, *p* = 0.002) (Fig. 3 and Table 4). Meanwhile, Glx level in mPFC was negatively correlated with the total PANSS-P score (Glx: *r* = −0.24, *p* = 0.011). However, the levels of Cr + PCr, GPC + PCh, and mI were not significantly correlated with the severity of pAVH (*p* > 0.05) (Table 4). No significant correlations were found between the levels of tNAA or Glx and the scores of PANSS-T, PANSS-P, or PANSS-N (*p* > 0.05). Similarly, the levels of tNAA and Glx were not significantly correlated with participants’ age, gender, education level, course of disease, and CPZ equivalent dosage, respectively (*p* > 0.05).

**Table 4:** Partial correlation analysis between the score of P3 hallucination item of PANSS and the type of metabolites.

Metabolite types	P3	
	*r*	*p*
tNAA	−0.24	0.014^*^
Glx (Glu + Gln)	−0.30	0.002^**^
Cr + PCr	0.02	0.85
GPC + PCh	−0.15	0.12
mI	−0.08	0.44

*P3* P3 hallucination item of PANSS, *tNAA* N-acetyl-aspartate(NAA) + N-acetyl-aspartyl-glutamate (NAAG), *Glx(Glu* + *Gln)* glutamate(Glu) + glutamine(Gln), Cr + PCr creatine + phosphocreatine, *GPC* *+* *PCh* glycerophosphocholine + phosphocholine, *mI* myo-inositol.

*p < 0.05; **p < 0.01.

## Discussion

To our knowledge, this is the first study to report differences in metabolite levels in the mPFC between patients with pAVH, between patients with non-AVH, and HC. In fact, most relevant studies that looked at the level of metabolites in the prefrontal cortex have made comparison between SCZ patients and HC, with no further subgroup comparisons within the SCZ group. To fill this gap, the present study aimed to evaluate the differences in the level of metabolites in the mPFC in patients with pAVH, patients with non-AVH, and HC, as well as the relationships between metabolite levels and the severity of pAVH.

After controlling for relevant covariates, we found that the tNAA level in mPFC of the pAVH group was lower than that in the non-AVH and HC groups, while there was no difference in the tNAA level in mPFC between the non-AVH and HC groups. Furthermore, the tNAA level in the mPFC was negatively correlated with the severity of pAVH. The above findings are consistent with our initial research hypotheses that the tNAA level in patients with pAVH would be lower than that in the other two groups. Although we found four studies investigating the relationship between the tNAA level in prefrontal cortex and symptoms of SCZ (including positive [17, 32] and negative [40, 41] symptoms), there was no study exploring the relationship between the tNAA level in mPFC and the severity of pAVH. Previous ^1^H-MRS studies of the frontal cortex of SCZ patients also showed inconsistent results. Some studies reported reduction in the level of tNAA in SCZ patients [41–44], while other studies reported no difference between SCZ patients and HC [32, 45, 46]. Despite the variety of methods, few studies made subgroup comparisons within the SCZ group, and most studies measured metabolite ratios, making comparison difficult [17]. It is worth noting that the results of this study were opposite to those of only one previous study in which the tNAA level in patients with non-AVH was lower than that in those with pAVH, and the tNAA level in DLPFC was positively correlated with the severity of pAVH [17]. This inconsistency in results might be attributed to clinical heterogeneity of the participants, the size of the sample and differing parameters, such as scanner model, field strength (1.5 or 3 or 7 Tesla), and voxel position.

Although NAA is the major contributor to the tNAA peak in 1H-MRS, 10% of the peak can be attributed to NAAG13. Because of the high spectral overlap of NAA and NAAG, it is difficult to segregate NAA and NAAG using MRS [47–49]. Therefore, tNAA—the sum of NAA and NAAG—was used as an evaluation index in this study. Since both NAA and NAAG are highly abundant in and relatively exclusive to neurons, abnormal levels of tNAA may reflect neuronal dysfunction. NAA is a major neuronal amino acid and also an important neuromaterial involved in the maintenance of axon–myelin integrity [26, 50]. NAA is involved in energy production and may reflect the functional state of neuronal mitochondria [28, 29]. Studies have found that reduced tNAA might be associated with neuronal degeneration or loss of dendritic pathology. Studies using models of psychotic symptoms, especially those based on ketamine (an *N*-methyl-d-aspartate receptor-NMDA-R—antagonist), have found that psychotic symptoms (including auditory hallucinations) could be caused by the binding of D2 receptors to induce DA release [51]. Some studies found that the metabotropic glutamate receptor type 3 (GRM3) allele that encodes type 2/3 metabotropic glutamate receptors (mGluR2/3) was associated with decreased levels of tNAA in the prefrontal cortex [52], which might be related to an imbalance of DA release in SCZ patients [53]. In addition, NAA is converted to NAAG by the enzymes NAAG synthetase I and II. NAAG selectively activates GRM3 and is converted to NAA by glutamate carboxypeptidases (GCPII/III) expressed on the outer surface of astrocytes [30]. It is worth noting that NAAG is likely to reduce the release of Glu and DA by activating the presynaptic mGluR2/3 receptor [54], and to reduce the cytotoxicity caused by excessive release of Glu and DA. This finding is indirectly supported by the present study.

Moreover, our findings emphasize the role of mPFC in the presence of pAVH and are in line with the cognitive model of AVH. Previous neuroimaging studies have shown that mPFC is an additional brain area related to hallucination in patients with SCZ [55–57]. Psychological fMRI studies revealed that mPFC is a core region of the default mode network, which is involved in self-monitoring [58]. And according to Simons et al. the anterior portion of mPFC is also involved in reality monitoring, i.e., distinguishing self-generated information from external information [59]. Another study reported that in a realistic monitoring task, SCZ patients with pAVH showed decreased activities in mPFC [60–62], which was associated with difficulty in distinguishing eventual hallucination experience from their own sensory activities [59, 63].

In this study, we also found that the Glx level in mPFC of the pAVH group was lower than those in the non-AVH and HC groups, while there was no difference in the Glx level in mPFC between patients with non-AVH and HC. Also, the Glx level in the mPFC was negatively correlated with the severity of pAVH. This is consistent with our hypothesis for this study, the result of our previous study that found reduced Glu level in the ACC in patients with unremitted SCZ, the results of a previous meta-analysis on the reduction of Glx in the frontal lobe of patients with SCZ [64], and the hypothesis of glutamatergic dysfunction in SCZ [24]. However, it is contrary to one study on the relationship between Glx and pAVH [2]. The inconsistency might be attributed to clinical heterogeneity in participants, including different grouping criteria, different sample size, and different research parameters. In the previous study, the duration of illness in the non-AVH group (9.5 years) was longer than that of the pAVH group (8.2 years) [2], while in the current study, the illness duration in the pAVH group (7.0 years) was higher than that in the non-AVH group (6.0 years). The findings of the current study appeared to support the finding that chronicity of SCZ is related to decreased level of Glx. Previous studies also revealed that the duration of SCZ was negatively correlated with the Glx level [65], which may also be one of the reasons for the different results of the two studies.

Since low magnetic fields distinguish poorly between the Glu and Gln signals, the sum of Glu and Gln (referred to as “Glx”) was used in the present study and in much of the literature [27, 49, 66]. Thus, while reading the following discussion of glutamatergic results in SCZ, one should take care in extrapolating Glx results to Glu. Glu is converted into Gln in astrocytes and Gln is used to synthesize Glu in neurons; this is the “glutamate–glutamine cycle” [11]. Glu is not only the most important excitatory neurotransmitter in the brain, but also an important regulator of neural pathways, and plays an indispensable role in the pathophysiology of SCZ [24, 66]. It has been reported that Gln, the Gln/Glu ratio, Glu or Glx levels in the mPFC are increased [67, 68] in individuals with prodromal SCZ with little or no use of antipsychotics [69], in those with deteriorated psychotic status [66], and in high-risk people who later developed SCZ [70]. These findings suggest that Glu release might increase in the early stage of SCZ, which might be related to the downstream effect of decreased NMDA-R function [71]. Such an increase in Glu may play a role in the decreased brain volume [72] and decreased nerve fibers detected at autopsy [73] in patients with early stage SCZ. In contrast, glutamatergic metabolites in the mPFC decreased when the patients were stabilized with antipsychotics or in the chronic phase [48, 64]. Studies have also pointed out that low-dose chronic phencyclidine (PCP) exposure can lead to the inactivation of extracellular Glu release [70] in the mPFC. However, the possible relationship between Glx reduction and pAVH cannot be separated from NMDA-R.

There are some limitations in this study. Firstly, the study is cross-sectional; therefore, follow-up visits are needed to further explore symptom development and prognosis, as well as the relationship between symptoms and metabolites in the brain. Secondly, only chronically ill patients with treatment-resistant symptoms were included, although there were no significant differences in the dosage of antipsychotics (CPZ equivalence per day) and illness duration between the patient groups. Having the dosage controlled, we were precluded from further investigation of the influence of drugs on the level of metabolites. In future works, greater efforts need to be made to explore the relationship between antipsychotics and the level of metabolites in untreated first-episode SCZ patients with pAVH.

## Conclusion

The present study has highlighted the relationship between pAVH and the level of metabolites in the mPFC, with the most notable result being that the tNAA and Glx levels were significantly lower in patients with pAVH compared with patients with non-AVH and HC. There were no differences in the tNAA and Glx levels between patients with non-AVH and HC. Our findings also showed that tNAA and Glx levels in mPFC were negatively correlated with the severity of pAVH in SCZ patients. Overall, this study suggested that reduced levels of tNAA and Glx are implicated in patients with pAVH, and improving the tNAA and Glx levels in mPFC might be clinically significant in the treatment for pAVH.

## Supplementary information

Supplementary material 1

Supplementary material 2

## References

[1] Aleman A, de Haan EH (1998). On redefining hallucination. Am J Orthopsychiatry.

[2] Ćurčić-Blake B, Bais L, Sibeijn-Kuiper A, Pijnenborg HM, Knegtering H, Liemburg E (2017). Glutamate in dorsolateral prefrontal cortex and auditory verbal hallucinations in patients with schizophrenia: a 1H MRS study. Prog Neuro-Psychopharmacol Biol Psychiatry.

[3] Dyck MS, Mathiak KA, Bergert S, Sarkheil P, Koush Y, Alawi EM (2016). Targeting treatment-resistant auditory verbal hallucinations in schizophrenia with fmri-based neurofeedback—exploring different cases of schizophrenia. Front Psychiatry.

[4] Liu W, Yu H, Jiang B, Pan B, Yu S, Li H (2015). The predictive value of baseline NAA/Cr for treatment response of first-episode schizophrenia: a 1H MRS study. Neurosci Lett.

[5] González J, Aguilar E, Berenguer V, Leal C, Sanjuan J (2006). Persistent auditory hallucinations. Psychopathology..

[6] Shergill SS, Murray RM, McGuire PK (1998). Auditory hallucinations: a review of psychological treatments. Schizophr Res.

[7] Tracy DK, Shergill SS (2013). Mechanisms underlying auditory hallucinations—understanding perception without stimulus. Brain Sci.

[8] Goghari VM, Harrow M, Grossman LS, Rosen C (2013). A 20-year multi-follow-up of hallucinations in schizophrenia, other psychotic, and mood disorders. Psychol Med.

[9] Sommer IE, Slotema CW, Daskalakis ZJ, Derks EM, Blom JD, van der Gaag M (2012). The treatment of hallucinations in schizophrenia spectrum disorders. Schizophr Bull.

[10] Shergill SS, Murray RM, McGuire PK (1998). Auditory hallucinations: a review of psychological treatments. Schizophr Res.

[11] Wu Q, Qi C, Long J, Liao Y, Wang X, Xie A (2018). Metabolites alterations in the medial prefrontal cortex of methamphetamine users in abstinence: a 1H MRS study. Front Psychiatry.

[12] Xu P, Chen A, Li Y, Xing X, Lu H (2019). Medial prefrontal cortex in neurological diseases. Physiol Genom.

[13] Landim RC, Edden RA, Foerster B, Li LM, Covolan RJ, Castellano G (2016). Investigation of NAA and NAAG dynamics underlying visual stimulation using MEGA-PRESS in a functional MRS experiment. Magn Reson Imaging.

[14] Chan KL, Saleh MG, Oeltzschner G, Barker PB, Edden R (2017). Simultaneous measurement of Aspartate, NAA, and NAAG using HERMES spectral editing at 3 Tesla. NeuroImage..

[15] Steinmann S, Leicht G, Mulert C (2014). Interhemispheric auditory connectivity: structure and function related to auditory verbal hallucinations. Frontn Hum Neurosci.

[16] Molina V, Sanchez J, Sanz J, Reig S, Benito C, Leal I (2007). Dorsolateral prefrontal N-acetyl-aspartate concentration in male patients with chronic schizophrenia and with chronic bipolar disorder. Eur Psychiatry.

[17] Psomiades M, Mondino M, Fonteneau C, Bation R, Haesebaert F, Suaud-Chagny M-F (2018). N-Acetyl-Aspartate in the dorsolateral prefrontal cortex in men with schizophrenia and auditory verbal hallucinations: a 1.5 T Magnetic Resonance Spectroscopy Study. Sci Rep.

[18] Wolf ND, Sambataro F, Vasic N, Frasch K, Schmid M, Schönfeldt-Lecuona C (2011). Dysconnectivity of multiple resting-state networks in patients with schizophrenia who have persistent auditory verbal hallucinations. J Psychiatry Neurosci.

[19] Jardri R, Pouchet A, Pins D, Thomas P (2011). Cortical activations during auditory verbal hallucinations in schizophrenia: a coordinate-based meta-analysis. Am J Psychiatry.

[20] Dollfus S, Alary M, Razafimandimby A. (eds). Speech processing and auditory hallucinations. (Springer, New York, 2013).

[21] Kubera KM, Sambataro F, Vasic N, Wolf ND, Frasch K, Hirjak D (2014). Source-based morphometry of gray matter volume in patients with schizophrenia who have persistent auditory verbal hallucinations. Prog Neuro-Psychopharmacol Biol Psychiatry.

[22] Scheinost D, Tokoglu F, Hampson M, Hoffman R, Constable RT (2019). Data-driven analysis of functional connectivity reveals a potential auditory verbal hallucination network. Schizophr Bull.

[23] Rowland LM, Kontson K, West J, Edden RA, Zhu H, Wijtenburg SA (2013). In vivo measurements of glutamate, GABA, and NAAG in schizophrenia. Schizophr Bull.

[24] Li J, Ren H, He Y, Li Z, Ma X, Yuan L (2020). Anterior cingulate cortex glutamate levels are related to response to initial antipsychotic treatment in drug-naive first-episode schizophrenia patients. Front Psychiatry.

[25] Klär AA, Ballmaier M, Leopold K, Häke I, Schaefer M, Brühl R (2010). Interaction of hippocampal volume and N-acetylaspartate concentration deficits in schizophrenia: a combined MRI and 1H-MRS study. Neuroimage..

[26] Eric P, Camilo FS, Francisco RM, Sofia C, Gladys GC, Pablo LO, et al. Elevated myo-inositol, choline, and glutamate levels in the associative striatum of antipsychotic-naive patients with first-episode psychosis: a proton magnetic resonance spectroscopy study with implications for glial dysfunction. Schizophr Bull. 2016;2:415–24. 10.1093/schbul/sbv118 PMC4753594 26320195

[27] Yoo SY, Yeon S, Choi CH, Kang DH, Lee JM, Na YS (2009). Proton magnetic resonance spectroscopy in subjects with high genetic risk of schizophrenia: Investigation of anterior cingulate, dorsolateral prefrontal cortex and thalamus. Schizophr Res.

[28] Port JD, Agarwal N (2011). MR spectroscopy in schizophrenia. J Magn Reson Imaging.

[29] Das TK, Dey A, Sabesan P, Javadzadeh A, Théberge J, Radua J, et al. Putative astroglial dysfunction in schizophrenia: a meta-analysis of 1H-MRS studies of medial prefrontal myo-inositol. Front Psychiatry. 2018;9:438. 10.3389/fpsyt.2018.00438 PMC6160540 30298023

[30] Long PM, Moffett JR, Namboodiri A, Viapiano MS, Lawler SE, Jaworski DM (2013). N-acetylaspartate (NAA) and N-acetylaspartylglutamate (NAAG) promote growth and inhibit differentiation of glioma stem-like cells. J Biol Chem.

[31] Martinez-Granados B, Brotons O, Martinez-Bisbal M, Celda B, Marti-Bonmati L, Aguilar E (2008). Spectroscopic metabolomic abnormalities in the thalamus related to auditory hallucinations in patients with schizophrenia. Schizophr Res.

[32] Sigmundsson T, Maier M, Toone BK, Williams SC, Simmons A, Greenwood K (2003). Frontal lobe N-acetylaspartate correlates with psychopathology in schizophrenia: a proton magnetic resonance spectroscopy study. Schizophr Res.

[33] An L, Li S, Wood ET, Reich DS, Shen J (2014). NAAG detection in the human brain at 7T by TE optimization and improved wiener filtering. Magn Reson Med.

[34] Sheehan DV, Lecrubier Y, Sheehan KH, Amorim P, Janavs J, Weiller E (1998). The Mini-International Neuropsychiatric Interview (M.I.N.I.): the development and validation of a structured diagnostic psychiatric interview for DSM-IV and ICD-10. J Clin Psychiatry.

[35] Kay SR, Fiszbein A, Opler LA (1987). The positive and negative syndrome scale (PANSS) for schizophrenia. Schizophr Bull.

[36] Benetti S, Pettersson-Yeo W, Allen P, Catani M, Williams S, Barsaglini A (2015). Auditory verbal hallucinations and brain dysconnectivity in the perisylvian language network: a multimodal investigation. Schizophr Bull.

[37] Andreasen NC, Carpenter WT, Kane JM, Lasser RA, Marder SR, Weinberger DR (2005). Remission in schizophrenia: proposed criteria and rationale for consensus. Am J Psychiatry.

[38] Kane J (1988). Clozapine in treatment-resistant schizophrenics. Psychopharmacol Bull.

[39] Provencher SW (1993). Estimation of metabolite concentrations from localized in vivo proton NMR spectra. Magn Reson Med.

[40] Callicott JH, Bertolino A, Egan MF, Mattay VS, Langheim FJP, Weinberger DR (2000). Selective relationship between prefrontal N-acetylaspartate measures and negative symptoms in schizophrenia. Am J Psychiatry.

[41] Tanaka Y, Obata T, Sassa T, Yoshitome E, Asai Y, Ikehira H (2006). Quantitative magnetic resonance spectroscopy of schizophrenia: relationship between decreased N-acetylaspartate and frontal lobe dysfunction. Psychiatry Clin Neurosci.

[42] Deicken RF, Zhou L, Corwin F, Vinogradov S, Weiner MW (1997). Decreased left frontal lobe N-acetylaspartate in schizophrenia. Am J Psychiatry.

[43] Ende G, Braus DF, Walter S, Weber-Fahr W, Soher B, Maudsley AA (2000). Effects of age, medication, and illness duration on the N-acetyl aspartate signal of the anterior cingulate region in schizophrenia. Schizophr Res.

[44] Bustillo JR, Lauriello J, Rowland LM, Thomson LM, Petropoulos H, Hammond R (2002). Longitudinal follow-up of neurochemical changes during the first year of antipsychotic treatment in schizophrenia patients with minimal previous medication exposure. Schizophr Res.

[45] Choe B-Y, Suh T-S, Shinn K-S, Lee C-W, Lee C, Paik I-H (1996). Observation of metabolic changes in chronic schizophrenia after neuroleptic treatment by in vivo hydrogen magnetic resonance spectroscopy. Investig Radiol.

[46] Fukuzako H, Takeuchia K, Hokazono Y, Fukuzako T, Yamada K, Hashiguchi T (1995). Proton magnetic resonance spectroscopy of the left medial temporal and frontal lobes in chronic schizophrenia: preliminary report. Psychiatry Res.

[47] Castellano G, Dias C, Foerster B, Li LM, Covolan R (2012). NAA and NAAG variation in neuronal activation during visual stimulation. Braz J Med Biol Res = Rev Bras PesquiMed Biol/Soc Brasileira Biofis [et al].

[48] Zhang Y, Li S, Marenco S, Shen J (2011). Quantitative measurement of N-acetyl-aspartyl-glutamate at 3 T using TE-averaged PRESS spectroscopy and regularized lineshape deconvolution. Magn Reson Med.

[49] Tang J, Joseph O, Alger JR, Shen Z, Johnson MC, London ED (2019). N-Acetyl and glutamatergic neurometabolites in perisylvian brain regions of methamphetamine users. Int J Neuropsychopharmacol.

[50] Kossowski B, Chyl K, Kacprzak A, Bogorodzki P, Jednorog K (2019). Dyslexia and age related effects in the neurometabolites concentration in the visual and temporo-parietal cortex. Sci Rep.

[51] Jardri R, Hugdahl K, Hughes M, Brunelin J, Waters F, Alderson-Day B (2016). Are hallucinations due to an imbalance between excitatory and inhibitory influences on the brain?. Schizophr Bull.

[52] Marenco S, Steele SU, Egan MF, Goldberg TE, Straub RE, Sharrief AZ (2006). Effect of metabotropic glutamate receptor 3 genotype on N-acetylaspartate measures in the dorsolateral prefrontal cortex. Am J Psychiatry.

[53] Bertolino A, Breier A, Callicott JH, Adler C, Mattay VS, Shapiro M (2000). The relationship between dorsolateral prefrontal neuronal N-acetylaspartate and evoked release of striatal dopamine in schizophrenia. Neuropsychopharmacology..

[54] Moffett JR, Ross B, Arun P, Madhavarao CN, Namboodiri AM (2007). N-Acetylaspartate in the CNS: from neurodiagnostics to neurobiology. Prog Neurobiol.

[55] Zmigrod L, Garrison JR, Carr J, Simons JS (2016). The neural mechanisms of hallucinations: a quantitative meta-analysis of neuroimaging studies. Neurosci Biobehav Rev.

[56] Allen P, Laroi F, McGuire PK, Aleman A (2008). The hallucinating brain: a review of structural and functional neuroimaging studies of hallucinations. Neurosci Biobehav Rev.

[57] Goghari VM, Sponheim SR, MacDonald AW (2010). The functional neuroanatomy of symptom dimensions in schizophrenia: a qualitative and quantitative review of a persistent question. Neurosci Biobehav Rev.

[58] Northoff G, Bermpohl F (2004). Cortical midline structures and the self. Trends Cogn Sci.

[59] Simons JS, Garrison JR, Johnson MK (2017). Brain mechanisms of reality monitoring. Trends Cogn Sci.

[60] Vinogradov S, Luks TL, Schulman BJ, Simpson GV (2008). Deficit in a neural correlate of reality monitoring in schizophrenia patients. Cerebral Cortex.

[61] Garrison JR, Fernandez-Egea E, Zaman R, Agius M, Simons JS (2017). Reality monitoring impairment in schizophrenia reflects specific prefrontal cortex dysfunction. NeuroImage.

[62] Subramaniam K, Ranasinghe KG, Mathalon D, Nagarajan S, Vinogradov S (2017). Neural mechanisms of mood-induced modulation of reality monitoring in schizophrenia. Cortex.

[63] Yanagi M, Hosomi F, Kawakubo Y, Tsuchiya A, Ozaki S, Shirakawa O (2020). A decrease in spontaneous activity in medial prefrontal cortex is associated with sustained hallucinations in chronic schizophrenia: an NIRS study. Sci Rep.

[64] Kubota M, Moriguchi S, Takahata K, Nakajima S, Horita N (2020). Treatment effects on neurometabolite levels in schizophrenia: a systematic review and meta-analysis of proton magnetic resonance spectroscopy studies. Schizophr Res.

[65] Liemburg E, Sibeijn-Kuiper A, Bais L, Pijnenborg G, Knegtering H, van der Velde J (2016). Prefrontal NAA and Glx levels in different stages of psychotic disorders: a 3T 1H-MRS study. Sci Rep.

[66] Coyle JT, Konopaske G (2016). Glutamatergic dysfunction in schizophrenia evaluated with magnetic resonance spectroscopy. JAMA Psychiatry.

[67] McQueen G, Sendt KV, Gillespie A, Avila A, Lally J, Vallianatou K (2021). Changes in brain glutamate on switching to clozapine in treatment-resistant schizophrenia. Schizophr Bull.

[68] Marsman A, van den Heuvel MP, Klomp DW, Kahn RS, Luijten PR, Hulshoff Pol HE (2013). Glutamate in schizophrenia: a focused review and meta-analysis of (1)H-MRS studies. Schizophr Bull.

[69] Ohrmann P, Siegmund A, Suslow T, Pedersen A, Spitzberg K, Kersting A (2007). Cognitive impairment and in vivo metabolites in first-episode neuroleptic-naive and chronic medicated schizophrenic patients: a proton magnetic resonance spectroscopy study. J Psychiatr Res.

[70] Amitai N, Kuczenski R, Behrens MM, Markou A (2012). Repeated phencyclidine administration alters glutamate release and decreases GABA markers in the prefrontal cortex of rats. Neuropharmacology..

[71] Field JR, Walker AG, Conn PJ (2011). Targeting glutamate synapses in schizophrenia. Trends Mol Med.

[72] Moghaddam B, Javitt D (2012). From revolution to evolution: the glutamate hypothesis of schizophrenia and its implication for treatment. Neuropsychopharmacology..

[73] Garey LJ, Ong WY, Patel TS, Kanani M, Davis A, Mortimer AM (1998). Reduced dendritic spine density on cerebral cortical pyramidal neurons in schizophrenia. J Neurol Neurosurg Psychiatry.

