# Supplementary material for: Metabolite differences in the medial prefrontal cortex in schizophrenia patients with and without persistent auditory verbal hallucinations: a 1H MRS study
Source: Transl Psychiatry. 2022 Mar 23;12:116. doi: 10.1038/s41398-022-01866-5 (PMC8943150; doi:10.1038/s41398-022-01866-5)
Supplement: Supplementary file 1 — Supplementary material 1 [file 41398_2022_1866_MOESM1_ESM.docx]

Supplementary Table 1 Gender differences in metabolites in mPFC of participants

| Metabolites | Male | Female | p |
| --- | --- | --- | --- |
| NAA | 4.88±1.66 | 4.98±1.21 | 0.67 |
| Glx(Glu+Gln) | 7.66±1.87 | 7.81±2.08 | 0.62 |
| GPC+PCh | 1.12±0.18 | 1.07±0.24 | 0.14 |
| Cr+PCr | 5.17±0.76 | 5.18±0.73 | 0.96 |
| mI | 4.36±1.71 | 4.53±1.21 | 0.45 |

*Note:* NAA: N-acetyl-aspartate; GPC+PCh: glycerophosphocholine + phosphocholine; mI: myo-inositol; Cr+PCr: creatine + phosphocreatine; Glx(Glu+Gln): glutamate(Glu) + glutamine(Gln).
