# Supplementary material for: Metabolite differences in the medial prefrontal cortex in schizophrenia patients with and without persistent auditory verbal hallucinations: a 1H MRS study
Source: Transl Psychiatry. 2022 Mar 23;12:116. doi: 10.1038/s41398-022-01866-5 (PMC8943150; doi:10.1038/s41398-022-01866-5)
Supplement: Supplementary file 2 — Supplementary material 2 [file 41398_2022_1866_MOESM2_ESM.docx]

Supplementary Table 2 List of a standard basis set of metabolites

| metabolites | |
| --- | --- |
| L-alanine | guanidinoacetate |
| aspartate | phosphocholine |
| creatine | L-lactate |
| phosphocreatine | myo-inositol |
| γ-aminobutyric acid | N-acetylaspartate |
| glucose | N-acetylaspartylglutamate |
| glutamate | scyllo-inositol |
| glutamine | taurine |
| glycerophosphocholine | -CrCH2 |
| lipids (Lip) (Lip09, Lip13a, Lip13b, Lip20) | macromolecules (MM) (MM09, MM12, MM14, MM17 and MM20) |
